# Use of Milled *Acanthocardia tuberculate* Seashell as Fine Aggregate in Self-Compacting Mortars

**DOI:** 10.3390/ma17184665

**Published:** 2024-09-23

**Authors:** Ágata González-Caro, Antonio Manuel Merino-Lechuga, Enrique Fernández-Ledesma, José María Fernández-Rodríguez, José Ramón Jiménez, David Suescum-Morales

**Affiliations:** 1 Área de Química Inorgánica, Universidad de Córdoba, E.P.S de Belmez, Avenida de la Universidad s/n, E-14240 Córdoba, Spain; q32gocaa@uco.es; 2 Área de Ingeniería de la Construcción, Universidad de Córdoba, E.P.S de Belmez, Avenida de la Universidad s/n, E-14240 Córdoba, Spain; ammlechuga@uco.es (A.M.M.-L.); efledesma@uco.es (E.F.-L.); p02sumod@uco.es (D.S.-M.)

**Keywords:** seashell aggregate, self-compacting mortar, durability properties, circular economy

## Abstract

This study focuses on the feasibility of using ground *Acanthocardia tuberculate* seashells as fine aggregates for self-compacting mortar production. The obtained results show a promising future for coastal industries as their use eliminates waste products and improves the durability of these materials. The use of *Acanthocardia tuberculate* recycled aggregate, in terms of durability, improves the performance of all mixes made with seashells compared to those made with natural sand, although it decreases workability and slightly reduces mechanical strength. Proper mix design has beneficial effects, as it improves compressive strength, especially when the powder/sand ratio is 0.7. Three replacement ratios based on the volume (0%, 50%, and 100%) of natural limestone sand with recycled fine aggregate from *Acanthocardia tuberculate* seashells, and three different dosages modifying the powder/sand ratio (0.6, 0.7, and 0.8), were tested. The fresh-state properties of each self-compacting mixture were evaluated based on workability. The mineralogical phases of the hardened mixtures were characterised using X-ray diffraction, thermogravimetry, and differential analyses. Subsequently, the mechanical and durability properties were evaluated based on the compressive and flexural strengths, dry bulk density, accessible porosity for water and water absorption, drying shrinkage, mercury intrusion porosimetry, and water absorption by capillarity. Therefore, the use of *Acanthocardia tuberculate* seashells in cement-based systems contributes to circular economy.

## 1. Introduction

Shellfish aquaculture has substantially increased worldwide in recent years from 1 million tons in 1950 to >87.5 million tons in 2020. Aquaculture and fishery waste is expected to reach 106 million tons by 2030 [1]. However, many seashells generated during the production process are not fully utilised due to their limited commercial value. These shells are normally dumped in landfills and have harmful effects on both the environment and human health [1,2,3,4]. For example, intensive oyster culture results in the accumulation of organic matter and hydrogen sulphide. Hydrogen sulphide is a byproduct of sulphate reduction resulting from the decomposition of organic matter. Most shells accumulate in landfills [5]. As the deposition of seashells increases, several alternatives have been proposed to minimise this waste, as they are natural materials whose main composition is CaCO_3_ in Vaterite and Aragonite forms [6,7]. Studies have used seashells as a filtering medium [8] to adsorb hydrogen sulphide from water onto shells [5], construction materials [1,9,10,11,12], and catalysts [13].

Regarding the construction sector, many studies have attempted to replace natural aggregates or cement with seashell sand or fillers in mortar and concrete manufacturing. Researchers have also investigated the mechanical properties and durability of mortar and concrete [4,14,15,16,17]. Tayeh et al. [6] reviewed the use of seashell fillers as cement partial replacements and concluded that they could be utilised as raw material for limestone Portland cement with a CaCO_3_ composition of >90%. Kuo et al. [11] replaced different amounts (5%, 10%, 15%, and 20%) of oysters, used as sand in mortars, and studied their mechanical properties. They concluded that the strength was not greatly affected for replacements up to 20%, but workability was lower with oyster aggregate. González-Caro et al. [2] studied the effects of an *Acanthocardia tuberculate* seashell (ATS) filler in self-compacting mortars and found that its compressive strength was slightly lower than that of reference mixtures (manufactured with natural sand). Liao et al. [18] also studied the influence of the usage of oyster shell sand on the mechanical and durability properties of mortars and concluded that the workability of the mortar was reduced; with seashell substitution up to 30%, the resistance improved by 9.6% compared with that of reference mortars without shells.

The main problem with seashells is related to the angular shape and porosity of the particles which in most cases leads to a decrease in the mechanical properties and consistency due to the higher water absorption of the shells. The advantage of shells is that they do not negatively affect the hydration of cement. Liao et al. [19] studied the effects of different oyster shell particle sizes on the physical and mechanical properties of mortars and concluded that the compressive strength decreased as the particle size increased. However, the characteristics of the mortar and concrete made with shells are satisfactory. Thus, they can contribute to the development of the circular economy as they help avoid shell waste accumulation in landfills.

However, the consumption of natural aggregates for mortar and concrete manufacturing has caused several parts of the world to (among others) restrict their extraction due to changes in the course of rivers, depletion of sand resources, and the negative impact on biodiversity [2,3]. Conventional aggregates are usually acquired through mining, a process that requires significant energy and results in environmental problems such as pollution and the depletion of natural resources. Consequently, there is increasing interest in the development of alternative materials for cement-based composites [20]. In 2020, global aggregate consumption was of 55 billion tons. This number is expected to double over the next 10 years. In addition, many forests have been destroyed by rapid urbanisation [21]. Therefore, the European Union is promoting a new paradigm of utilising waste as a secondary raw material (SRM) in various production processes to advance the circular economy model [22].

Although the use of waste as a SRM can optimise resource use, recover waste, and reduce environmental emissions, these materials often present uncertainties regarding their mechanical strength and durability. To ensure reliability, it is necessary to assess durability against degradation. Therefore, extensive studies on the hydration processes, material microstructure, and transport mechanisms within the cement matrix are required [23].

Self-compacting concrete (SCC) is an advanced construction material that improves conventional concrete (CC) by using more cement and mineral additions and fewer coarse aggregates. This composition allows for easier placement without inducing vibration and results in better surface quality, higher strength, and greater durability. Consequently, SCCs are extensively used in various construction applications. Billberg [23] contended that the rheology of concrete can only be perfected if the mortar phase is tailored to achieve optimal rheological performance. Thus, the performance of SCC is largely determined by the performance of the self-compacting mortar phase (SCM). Nepomuceno et al. [24] and da Silva and de Brito [25] studied the SCM phase to forecast the SCC characteristics. Furthermore, the SCC is costlier than the CC. To address this limitation, supplementary materials are used to replace partially fine materials, thereby reducing costs and enhancing the durability of concrete.

To solve the problem of shell accumulation in landfills in the construction sector and the use of natural aggregates, a good alternative is to replace these natural aggregates with crushed seashells. To the best of our knowledge, to date, no research has been conducted using the ATS in SCM.

This study aims to investigate the effect of substituting calcareous natural aggregate (NA) with recycled aggregate from ATS to test its viability in SCM production. ATS is a cockle shell species, with 20–24 well-marked ribs and numerous irregular fine concentric lines.

The results indicate good performance in terms of both durability and strength using ATS as an aggregate. This research helps prevent the deposition of ATS in landfills and reincorporates it into the construction industry as an SRM, which aligns with the new circular economy model of the European Union. This comprehensive research using ATS as an aggregate will support continued innovation in the construction sector, specifically in SCM.

## 2. Materials and Methods

### 2.1. Materials

In this study, two types of natural aggregate were used for mortar (SCM) production: natural aggregate 0/3 (NA-0/3) and 0/6 (NA-0/6). The aggregates were obtained from a quarry in Córdoba, Spain. According to the manufacturer, both sands are calcareous in nature, and a limestone filler (LF) from Taljedi, S.L., was used. Ordinary Portland cement (OPC) was used in all experimental studies, which possesses a minimum compressive strength of 42.5 MPa according to the EN 197-1 standard, was supplied by Votorantim Cimentos (Córdoba, Spain) [26]. To achieve a self-compacting mixture, a superplasticiser (Sp) composed of polyaryl-ether polymers with a density of 1058 kg/m^3^ was supplied by BASF Española SL.

ATS samples were obtained from a company located in Málaga, Spain. To obtain recycled fine aggregates from ATS waste, a subsequently described treatment method was applied.

To remove any organic material and sulphate salts, ATS samples were cleaned by washing with water and then dried in an oven (135 °C for 32 min) [7]. The ATS samples had sulphate concentrations equal to 0.05% and 0.01% by mass (UNE-EN 1744-1) [27] before and after the washing process, respectively. These values fell below the threshold outlined in the Structural Code employed in Spain [28].

**Figure 1:**
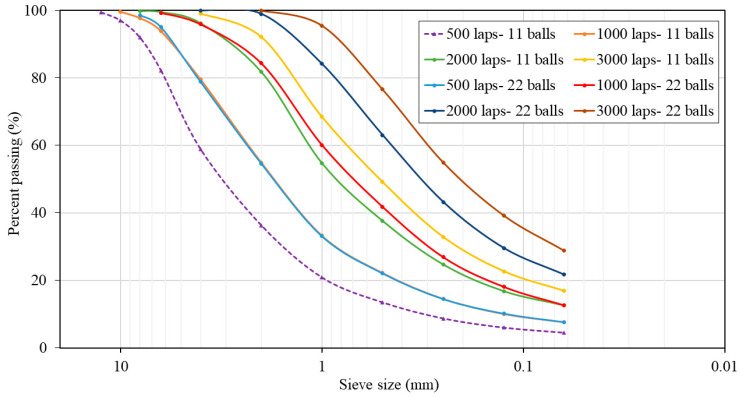
Particle size distributions of *Acanthocardia tuberculate* seashells (ATSs) with different milling processes.

To replace NA-0/3 and NA-0/6 with 0%, 50%, and 100% of ATS recycled fine aggregates, both materials should have similar particle size distributions. For this purpose, a Los Angeles machine was used as a ball mill to grind the seashells. Eight milling cycles were performed by varying the number of laps (500, 1000, 2000, and 3000) and using 11 or 22 standardised steel balls. The particle size distribution curves for the various milling processes were determined in accordance with UNE EN 933-1:2012 [29] standard, as shown in Figure 1. This procedure was carried out in a previous study by the authors [2].

**Figure 2:**
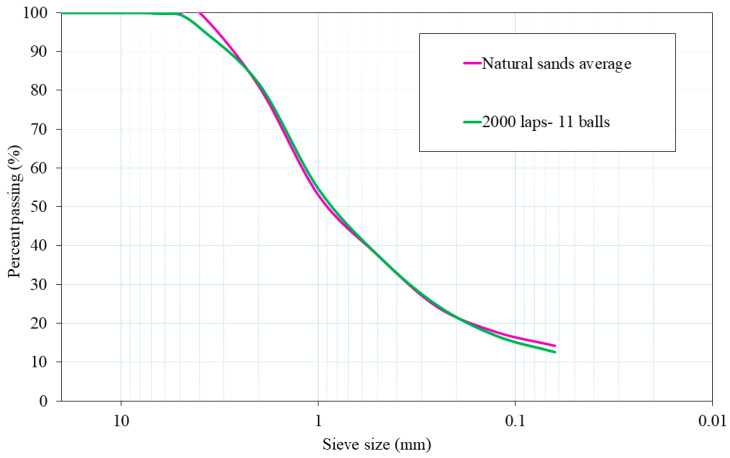
Particle size distributions of natural sands average (NA-0/3 and NA-0/6) and ATS aggregate chosen for this research.

**Figure 3:**
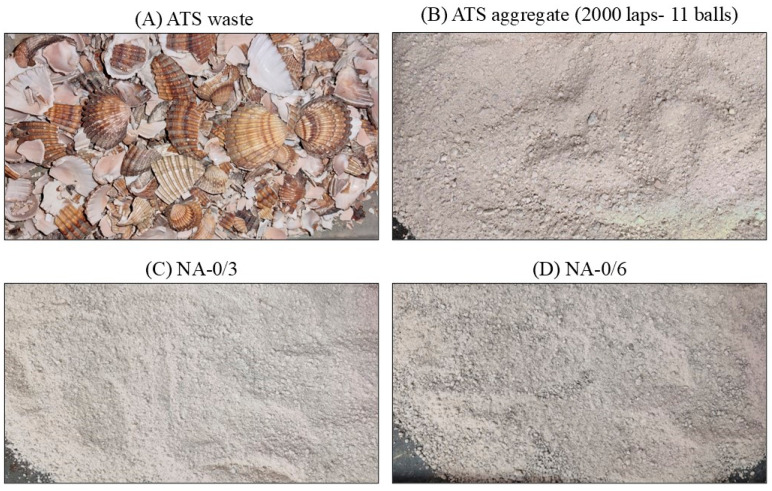
Images of the aggregates used for SCM production. (**A**) ATS waste, (**B**) ATS aggregate (2000 laps-11 balls), (**C**) NA-0/3, and (**D**) NA-0/6.

The chosen milling procedure was 2000 laps and 11 balls because its particle size distribution was the most similar to the average particle size distribution of both natural aggregates. Figure 2 shows plots of the mixtures of NA-0/3 and NA-0/6, as well as ATS-recycled fine aggregates chosen for this study. Figure 3 shows the characteristics of the aggregates used.

The skeletal density and water absorption were measured according to UNE-EN-1097-6:2009 [30]. Table 1 lists the basic physical parameters of all the aggregates used for SCM production.

### 2.2. Self-Compacting Mortar Design

A self-compacting mortar design was developed based on both the Nepomuceno method [24] and experimental and iterative processes. The method classifies cement and mineral additions as “powders”. The sand was considered as “fine aggregate”. Therefore, CEM and LF were considered as “powder materials”, and NA-0/3, NA-0/6, and ATS aggregate were considered as “fine aggregates”. Table 2 lists the nomenclature, mixing proportions (kg/m^3^), and self-compactability parameters of each SCM. Self-compactability parameters are based on the absolute volume proportions of the following ratios: (i) volume of powder materials and fine aggregate (Vp/Vs), (ii) volume of water and powder materials (Vw/Vp), and (iii) mass percentage of the superplasticiser and powder materials (Sp/p %). The Vw/Vp (0.85) and w/c (0.49) ratios were the only parameters that remained constant throughout the study.

Reference mortars were manufactured using 50% NA-0/3 and 50% NA-0/6. Replacement volumes of 50% (25% NA-0/3, 25% NA-0/6, 50% ATS aggregate) and 100% ATS aggregate were tested in three reference mixtures, namely Ref-1, Ref-2, and Ref-3.

Depending on the value of the ratio (Vp/Vs), groups 1, 2, and 3 were tested for values of 0.6, 0.7, and 0.8. In all the groups, the CEM/LF ratio of 1.63 was maintained. In addition, for group 3, an Sp/p ratio value of 0.5 was used to obtain a self-compacting mixture.

The appropriate values that met the self-compactability conditions were those that fulfilled the flow requirements experimentally measured using two tests: slump flow and V-funnel; the first obtained the relative spread area (Gm) and the second the relative flow velocity (Rm). The value of Gm is given by Equation (1), whereas Equation (2) shows the value of Rm.

(1) $$Gm={\left(\frac{Dm}{D_{0}}\right)}^{2}-1$$

(2) $$Rm=\frac{10}{t}$$

where Dm represents the spread diameter in millimetres, D_0_ represents the initial diameter at the base of the cone (in mm), and t represents the flow time (in s). According to the EFNARC guidelines [31], the diameter of the cone used in a slump flow test should be 100 mm, and the V-funnel test should have a height of 300 mm.

The kneading procedure was as follows: the solid materials (dried at 105 °C for 24 h) were homogenised at a low speed for 30 s in a standard mortar mixer. Water was then added together with the superplasticiser and mixed for 6 min at a low speed. Subsequently, the mixing process was stopped for 2 min to ensure that all constituents were blended. Finally, the mixture was mixed again for 2 min at a low speed.

Prismatic moulds with dimensions of 40 × 40 × 160 mm^3^ were manufactured for each type of SCM without the required compaction and vibration energy. The specimens were unmoulded after 24 h and cured in a climatic chamber at a relative humidity of 95 ± 5% and a temperature of 20 ± 2 °C for 28 d.

### 2.3. Test Methods

To examine the chemical composition of the raw materials (CEM, NA-0/3, NA-0/6, ATS aggregate, and LF), X-ray fluorescence spectroscopy (XRF) was performed using a ZSX Primus IV Rigaku instrument with a potency rating of 4 kW. X-ray diffraction (XRD) was employed to identify the mineral phases in the raw materials and hardened mortars. A Bruker D8 Discover A25 instrument with CuKα (λ = 1.54050 Å, 40 kV, 30 mA) was employed to acquire diffraction patterns by scanning the goniometer from 10° to 70° (2θ) at a rate of 0.006 θ min^−1^. Thermogravimetric (TGA) and differential thermal analysis (DTA) were conducted on the raw material and hardened mortar samples, utilising a Setaram Setsys Evolution 16/18, with testing temperatures spanning from 25 °C to roughly 1000 °C, a heating rate of 5 °C_min^−1^ with testing temperatures spanning from 25 °C to roughly 1000 °C and introducing a sample mass between 30-40 mg.

The mechanical properties of the mixtures were evaluated by testing their flexural and compressive strengths after curing at 28 d in a climatic chamber at 20 ± 2 °C with a relative humidity of 95 ± 5%, according to UNE-EN 1015-11:2020 [32]. For the quantification of flexural strength, three specimens of each mixture were evaluated using prismatic samples with the sizes of 40 mm × 40 mm × 160 mm, while six semi-prismatic samples with the sizes of 40 mm × 40 mm × ≈80 mm were used for the compressive strength.

The dry bulk density, water absorption by immersion, and accessible porosity of water were determined according to UNE-EN 83980 [33] after 28 d of curing. Mercury intrusion porosimetry (MIP) tests were performed on all specimens to determine the volume and distribution of pores. After 28 d, the samples were heated at 100 °C until their weights stabilised. A section with a volume of approximately 1 cm^3^ was obtained from the central region of each specimen. The equipment used was a Quantachrome Poremaster 60GT at a pressure range of 0.0014–414 MPa [34], and the shrinkage and mass loss were realised according to UNE 83831:2021 [35] at 1, 7, 14, 28, 61, and 91 d. For the shrinkage measures, an IBERTEST Universal Shrinkage Meter, ref. 210-102229 was used. It must be able to measure a minimum length of the specimen ±5 mm, with an accuracy of ±0.001 mm. A calibration bar is used which must be 160 mm long. The same length of the moulds was used to manufacture the specimen. Water absorption by capillarity was determined according to UNE-EN 1015-18:2003 [36] at 28 d of curing.

## 3. Results and Discussion

### 3.1. Characterisation of Raw Materials

Table 3 summarises the chemical compositions of the raw materials analysed using XRF. CaO was the major component in all the raw materials (CEM, NA-0/3, NA-0/6, ATS aggregate, and LF). The CaO content found in CEM was 70.03% followed by the content of SiO_2_ (15.58%); lower contents were documented for Al_2_O_3_ (3.73%), SO_3_ (4.79%), and Fe_2_O_3_ (2.44%) [37,38]. The NA CaO contents (98.31% and 60.44% for NA-0/3 and NA-0/6, respectively) are consistent with their calcareous nature. This could be one of the factors indicating the appropriateness of the replacement of NAs with ATS aggregates. The MgO content was greater in NA-0/6 (37.98%) than in NA-0/3 (0.88%). The CaO content of ATS aggregates was 96.81%. The amount of CaO depends on the type of shell and drying temperature [2,16]. Other minor composition percentages for ATS aggregates were SiO_2_ (1.25%), Al_2_O_3_ (0.15%) and Fe_2_O_3_ (0.11%). Although the chemical composition varies depending on the type of shell, the main component identified by other authors using XRF is CaO [10,14,39,40,41]; hence, the replacement of NA with ATS aggregates is justified.

**Figure 4:**
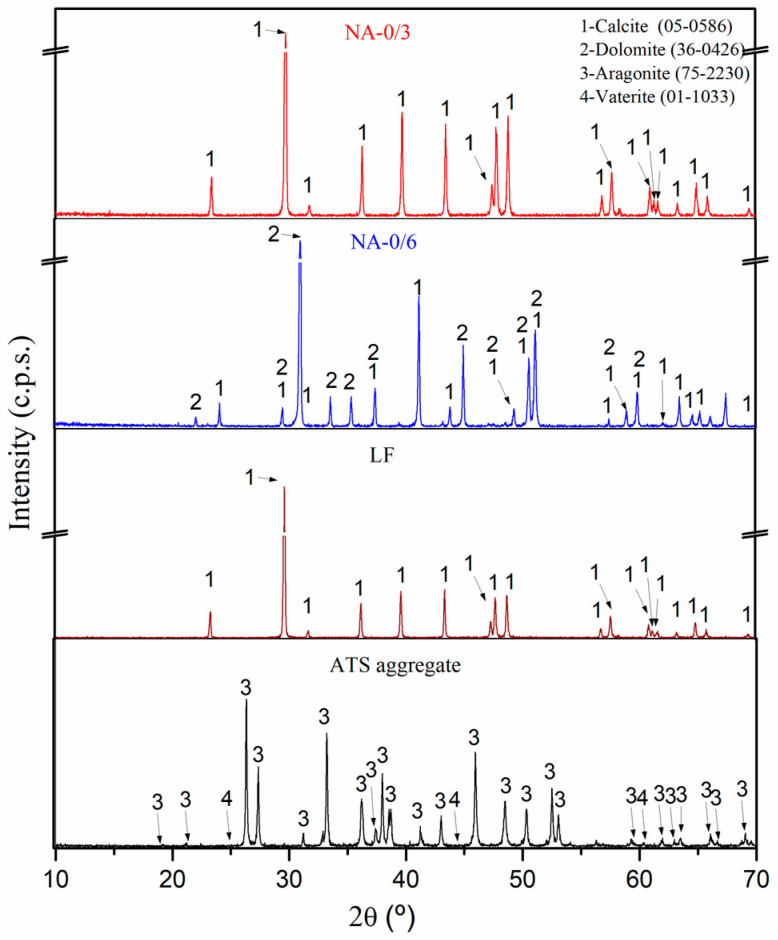
X-ray diffraction patterns characterisation of raw materials (NA-0/3, NA-0/6, LF, and ATS aggregate).

Figure 4 shows the mineralogical compositions of the raw materials (NA-0/3, NA-0/6, LF, and ATS aggregates) obtained using XRD. The main phase of NA-0/3 and NA-0/6 was calcite (CaCO_3_) (05-0586) [42]. The dolomite (CaMg(CO_3_)_2_) (36-0426) [42] phase was found in NA-0/6, as verified by XRF (Table 3), and was one of its main components. Similar results were reported in other studies which involved similar aggregates [2]. The main phases identified in the ATS aggregates were aragonite (75-2230) [41] and vaterite (01-1033) [42]. Both phases are calcium carbonate (CaCO_3_) polymorph calcite. Several authors [43,44] have detected aragonite in seashell powder and ATS. The calcite (CaCO_3_) (05-0586) [42] phase was also the main phase found in LF.

**Figure 5:**
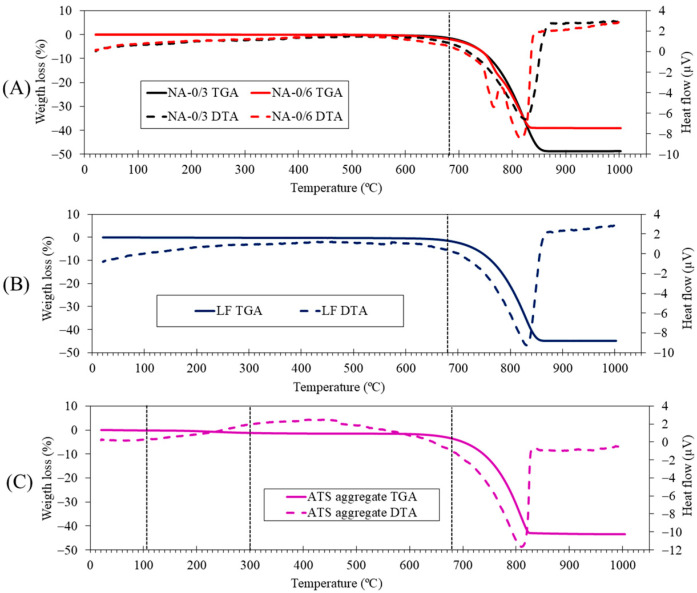
Thermogravimetric and differential thermal analysis (TGA-DTA) of raw materials: (**A**) NA-0/6 and NA-0/3, (**B**) LF, and (**C**) ATS aggregate.

Figure 5 shows the TGA and differential analysis (TGA-DTA) of raw materials. An endothermic peak can be observed at 830 °C in the LF (A) case which corresponds to the decomposition of calcite. This agrees with the calcite phase observed in the XRD results (Figure 4). In the case of NA-0/3 (B), an endothermic peak is observed at approximately 830 °C; this corresponds to the decomposition of calcite, which is in agreement with the calcite phase found by XRD (Figure 4) and the main oxide (CaO) found by XRF (Table 3). In the case of NA-0/6 (B), in addition to the endothermic peak corresponding to the decomposition of calcite at 830 °C, another endothermic peak is observed at 760 °C that corresponds to the decomposition of dolomite (CaMg(CO_3_)_2_) identified by XRD (Figure 4). The presence of Mg was indicated by XRF (Table 3). These results are consistent with those of previous studies [45,46,47]. Several stages were observed in the case of the ATS aggregate (C): (i) moisture loss occurs from 25 °C up to 105 °C, (ii) loss of more volatile organic matter occurs from 105 °C to 300 °C, and (iii) loss of more resistant organic matter occurs from 400 °C to 500 °C; (iv) between 600 °C and 1000 °C, an endothermic peak is observed (at 820 °C) owing to the decomposition of calcium carbonate, according to Barros et al. [48] and Mohamed et al. [49] who, respectively, studied mussel and cockle shells. Additionally, in the DTA curve, several thermal effects are observed, which are associated with the transformation of vaterite (T > 456 °C) and aragonite (400–494 °C) in calcite; these effects are endothermic for aragonite and exothermic for vaterite [50].

### 3.2. Fresh Properties of Mixtures

Table 4 lists the fresh properties of the SCM mixtures studied in this study through the self-compacting parameters, Gm and Rm, as explained in Section 2.3. An experimental and iterative process (described by Nepomuceno [24] and according to the specifications of EFNARC [31]) was executed, wherein the admissible interval for all the tested mixtures were from 4.29 to 7.41 for Gm, and from 0.83 to 2.50 s^−1^ for Rm, to meet self-compactability parameters. The obtained results were satisfactory as fresh SCM mixtures showed no segregation and bleeding.

There was a minor decrease in consistency (Gm) and fluidity (Rm) as the percentage of ATS aggregates increased. This was due to the angular and irregular shape of the ATS, as previously observed using scanning electron microscopy [2]. This irregular shape makes the ATS aggregate behave similarly to the coarse aggregate in self-compacting concrete, appearing to have a drier consistency. Similar results were obtained by Brahim et al. [51] who explained that natural aggregates have a spherical shape which increases the fluidity of the mortar. Several studies have related irregular particle shapes and surface areas to decrease in consistency and fluidity [2,4,51,52,53]. Seo-Eun et al. [20] studied the flowability of mortars with different types of shells as aggregates (cockles and oysters) at the substitution percentages of 20%, 30%, and 40%, and concluded that flowability decreased as the percentage of substitution increased, mainly due to the water absorption of the shells compared with natural sand.

The use of superplasticisers is a good alternative for avoiding dry consistency. In the SCM, the amount of superplasticiser was added according to the demand of each mixture.

### 3.3. Hardened Properties of the Mixtures

#### 3.3.1. Characterisation of Hardened Mortar

**Figure 6:**
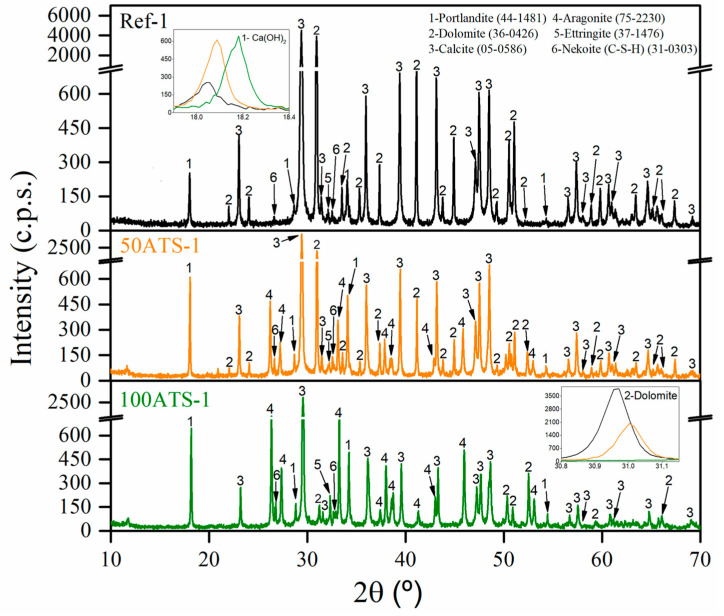
X-ray diffraction patterns of group-1 mixtures.

Figure 6 shows the XRD patterns of the group-1 SCM mixtures (Ref-1, 50ATS-1, and 100ATS-1) obtained after 28 d of curing. The phases formed in the group-2 and -3 mixtures were the same; therefore, it is unnecessary to include these graphs. The main phases identified in all the reference mixtures were calcite (CaCO_3_) (05-0586) and dolomite (CaMg(CO_3_)_2_). Both phases originated from NA-0/3 and NA-0/6 and through the use of XRD (Figure 4); accordingly, it was possible to identify that the main compounds were calcite and dolomite. Nekoite (Ca_3_Si_6_O_12_(OH)_6_·5H_2_O) (31-0303), portlandite [Ca(OH)_2_] (44-1481), and ettringite (Ca_6_Al_2_(SO_4_)_3_(OH)_12_·26H_2_O) (37-1476) phases, also named C-S-H, were also confirmed. This was attributed to the reactions that occurred in the cement [2,45,52]. In addition to the phases mentioned above for the 50ATS and 100ATS mixtures, the aragonite (CaCO_3_) (75-2203) phase was identified, which is in accordance with the phase found in the XRD pattern of the raw material (Figure 4). This phase has also been identified by other researchers who studied seashells in mortar or concrete [2,43,54].

The XRD technique approximately indicates the amount of each phase and is related to its intensity appearing on the y-axis. As shown in the inset of the portlandite (Ca(OH)_2_) phase, zooming between the 2θ values of 17.9° and 18.4° (lines black, yellow, and green lines for Ref, 50ATS, and 100ATS, respectively) shows that the amount increased following the substitution of NA by the ATS aggregate for all groups of mixtures.

**Figure 7:**
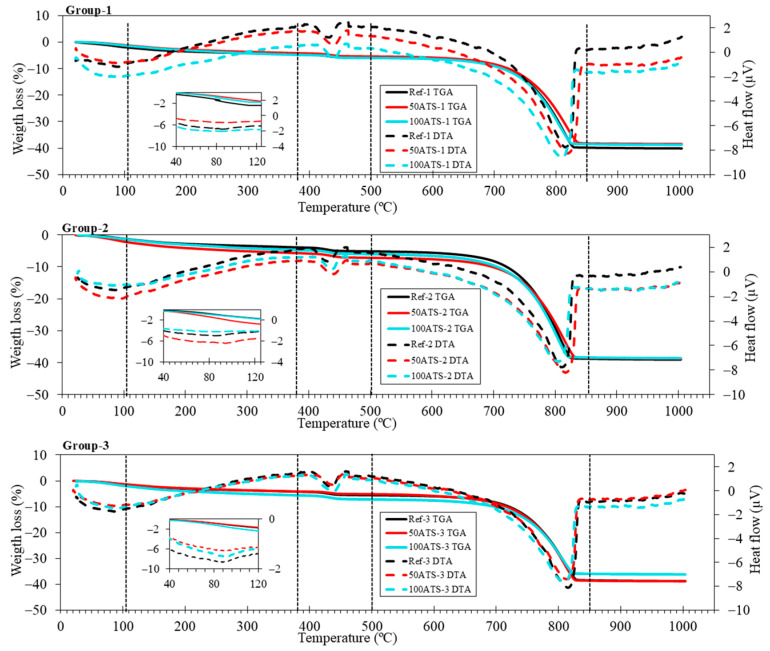
TGA (solid lines) and DTA (dotted lines) of the three studied SCM groups.

Figure 7 shows the TGA-DTA results of the three groups of studied SCMs. TGA was performed to quantify the presence of hydration products in the SCM. Five stages were differentiated in all the groups. The first stage was from 25 °C up to 105 °C and corresponded to water loss and the partial dehydration of ettringite [55]. The insets of all groups show an endothermic peak which also reflects the loss of these hydration products. The second stage was from 105 °C to 380 °C, wherein the decomposition of hydrated calcium silicates (C-S-H), aluminates, and ettringite took place [56]. The third stage was from 380 °C to 500 °C, which was due to the dehydroxylation of calcium hydroxide, also called portlandite (Ca(OH)_2_) [57,58], as observed in the XRD outcomes (Figure 6). The fourth stage was from 500 °C to 850 °C and was due to the loss of calcium carbonate (CaCO_3_) [55], which was formed during the cement hardening process and due to the raw materials used. The fifth (last) stage occurred between 850 °C and 1000 °C and corresponded to the removal of OH residuals.

The XRD results are in accordance with the weight loss calculated using TGA-DTA (as shown in Table 5), which indicates that the weight loss corresponding to the temperature ranges is related to the mineralogical phases. The first range (from 25 °C to 105 °C) corresponded to water loss. The second range (from 380 °C to 470 °C) was associated with the decomposition of the portlandite phase, formed during the setting process. In all SCM groups, there was an increase in the formation of portlandite as the replacement of seashell aggregates by natural sand increased. Wang and Liu [43] obtained a similar result, indicating that this increase in portlandite was due to the physical function of the seashell due to its irregular shape and complexity. The increase in portlandite may be due to the irregularity of the shell surface which acted as a nucleation site, favouring the formation of hydration products. The weight losses in the range of 850 °C–1000 °C were lower for all SCMs with ATS aggregates compared with the Ref; this means that the formation of carbonates is lower for SCM manufactured with the ATS aggregate. The dolomite (CaMg(CO_3_)_2_) inset shows that, with 100ATS, the amount of this phase decreases because this mixture does not contain NA-0/6 aggregates.

#### 3.3.2. Mechanical Strength

**Figure 8:**
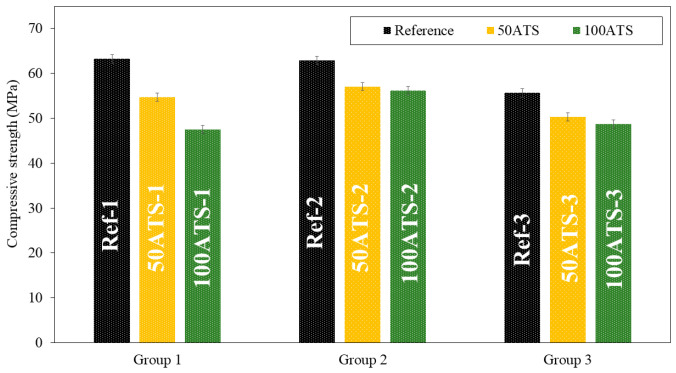
Compressive strengths of the three groups of studied mixtures (Ref, 50ATS, and 100ATS).

**Figure 9:**
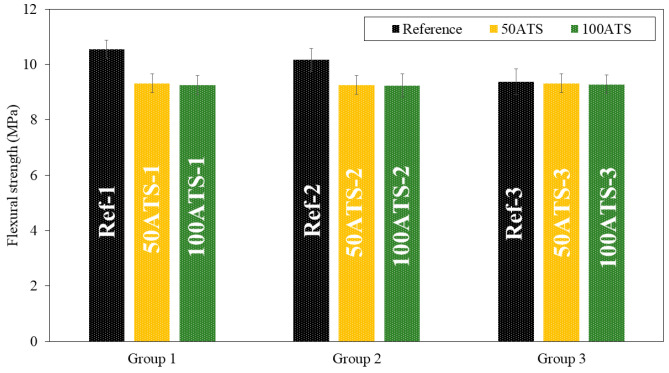
Flexural strengths of the three groups of studied mixtures (Ref, 50ATS, and 100ATS).

Figure 8 and Figure 9, respectively, show the compressive and flexural strengths for all the groups of mixtures studied after 28 d of curing. The reference mixtures (Ref-1, Ref-2, and Ref-3) show that the mean compressive and flexural strengths decrease slightly as the Vp/Vs ratio increases. A decrease in the compressive strength was observed as the ATS content increased. In group 1, these decreases were of 13.51% (Ref-1 vs. 50ATS-1) and 24.87% (Ref-1 vs. 100ATS-1). In group 2, the decreases were of 9.28% (Ref-2 vs. 50ATS-2) and 10.76% (Ref-2 vs. 100ATS-2), and in group 3, these were of 9.56% (Ref-3 vs. 50ATS-3) and 12.37% (Ref-3 vs. 100ATS-3). Therefore, as the replacement of the ATS increased, the compressive strength decreased.

The maximum loss in compressive strength was of 24.87% for mortars made with 100ATS when the Vp/Vs ratio was 0.6. The smallest decrease in strength for mortars made with 100ATS was only of 10.76% when the Vp/Vs ratio was 0.7. Therefore, the proportion of powders and aggregates affects the strength of self-compacting mortar.

Zhang et al. [17] studied the mechanical properties of foam concrete made of Babylonia areolata seashells, a sea snail species. They concluded that the compressive strength decreased as the seashell waste increased (from 35.8 MPa to 9.4 MPa); this was because, although the seashell exhibited solid characteristics, its strength was still lower than that of crushed stone. Suarez et al. [44] studied the mechanical properties of mortar containing ATS as a partial aggregate replacement and found that the compressive and flexural strengths decreased with increasing ATS replacements. They attributed this phenomenon to the high water absorption by the shells; this led to a decrease in the water/cement ratio and incomplete hydration of the cement. It is noteworthy that this is the only study conducted on this type of shell. Martínez-García et al. studied the behaviour of mussel shells as aggregates in concrete [7]. They made concrete by substituting natural limestone sand and gravel with mussel shell sand and gravel, and they agreed with the higher water absorption of seashells and the irregular shape of particle shells, which caused entrapped air in concrete and a decrease in compression. Seu-Eun et al. [20] studied the effects of oyster, cockle, and murex seashells as aggregates in cement-based composites with 20%, 30%, and 40% of substitution of natural sand by waste seashell sand and concluded that compressive strength decreased as the replacement ratio increased in all types of waste seashell aggregates. This phenomenon is attributed to the surface of the shell.

Therefore, the decrease in compression was mainly due to the higher water absorption and irregular shapes of the seashell particles [4,16,19,59]. This is in agreement with the results obtained for the fresh properties of the mixtures (Table 4) in which decreases in the consistency and fluidity of the mixtures were obtained by increasing the number of ATS aggregates. It should be noted that (to the best of our knowledge) no studies have reported the use of ATS as an aggregate in self-compacting mortars.

Figure 9 shows the flexural strength. In group 1, there were decreases of the orders of 11.66% (Ref-1 vs. 50ATS-1) and 12.23% (Ref-1 vs. 100ATS-1); in group 2, there were decreases of the orders of 8.86% (Ref-2 vs. 50ATS-2) and 9.06% (Ref-2 vs. 100ATS-2); and in group 3, there were decreases of the orders of 0.64% (Ref-3 vs. 50ATS-3) and 1.07% (Ref-3 vs. 100ATS-3).

#### 3.3.3. Dry Bulk Density, Accessible Porosity for Water, and Water Absorption

**Figure 10:**
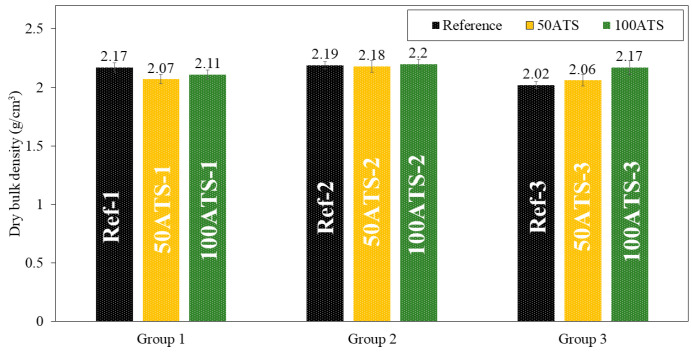
Dry bulk densities (g/cm^3^) of the three groups of studied mixtures (Ref, 50ATS, and 100ATS).

**Figure 11:**
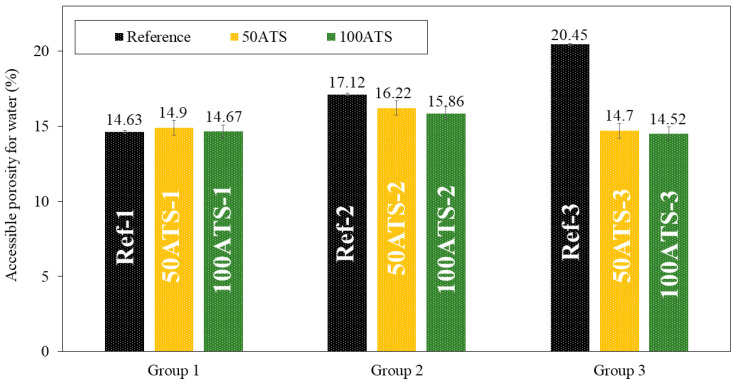
Accessible porosities for water (%) in the three studied groups of mixtures (Ref, 50ATS, and 100ATS).

**Figure 12:**
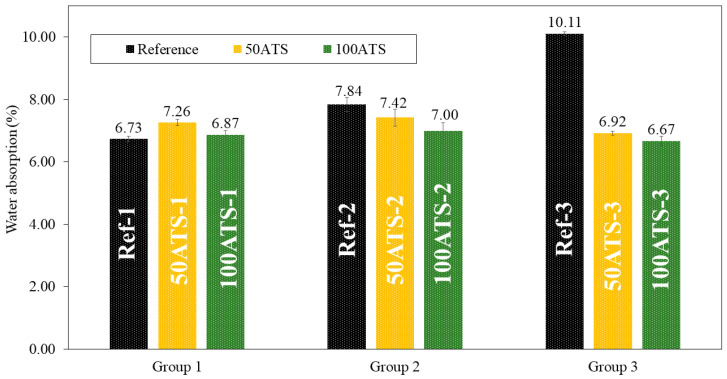
Water absorptions (%) in the three groups of studied mixtures (Ref, 50ATS, and 100ATS).

The dry bulk density (Figure 10), accessible porosity of water (Figure 11), and water absorption (Figure 12) of the hardened mortar were determined after 28 d of curing.

The dry bulk density (g/cm^3^) of the mixtures of group 1 decreased to replace the 50ATS and 100ATS aggregates by 4.61% (Ref-1 vs. 50ATS-1) and 2.76% (Ref-1 vs. ATS-1), respectively. In group 2, the densities maintained very similar values: 2.19, 2.18, and 2.2 g/cm3 for Ref-2, 50ATS-2, and 100ATS-2, respectively. In group 3, an increase in density occurred to replace 50ATS and 100 ATS by 1.98% (Ref-3 vs. 50ATS-3) and 7.43% (Ref-3 vs. 100ATS-3). Overall, the densities were very close to those of the reference mixtures, except for 100ATS-3, which may be related to the greater ratio Vp/Vs (Table 2), as increasing the amount of powder materials produced a densification of the mixture. In group 2, the number of fines was balanced by the number of coarse particles; hence, the densities were very similar. However, the irregular shapes of seashells caused the formation of voids which reduced the density [2,7]. Martínez-García et al. studied the substitutions of natural sand with mussel shell sand in non-structural concrete at the rates of 25%, 50%, 75%, and 100%. Reductions of 5% and 10% in densities were, respectively, obtained when the percentages of substitution of mussel shell aggregate increased from 25% to 100%. This phenomenon is explained by the flaky shape of the particles, which causes air to enter.

The accessible porosity for water (APW), shown in Figure 11, was similar for all group 1 and 2 mixtures. In the mixtures of group 3, decreases in accessible porosity of 28.12% (Ref-3 vs. 50ATS-3) and 28.99% (Ref-3 vs. 100ATS-3) occurred with respect to the reference. These results are consistent with those obtained for the density (Figure 10) and water absorption (Figure 12). The decrease in the porosity of the group 3 mixtures may increase the Vp/Vs ratio, which in turn increases the amount of powder materials, thus producing a packing effect [2,16]. APW typically increases with the percentage of shell replacement; however, in SCM, owing to the amount of powder materials, these values are counterbalanced. Safi et al. [59] studied the porosity of SCM made with waste seashells as aggregates and found that, as the percentage of substitution increased (up to 100%), the porosity decreased. This can be explained by the angular shape of the seashells, which promotes contact between particles. When <50% in volume of the oyster seashell is replaced, a combination of sand (which has a rounded shape) and crushed seashells (which have angular shapes) can create voids, leading to increased porosity and water absorption. When the replacement was of 100% in volume crushed shells, the contact among particles became optimal and the distribution of crushed shell grains was uniform. This behaviour can occur in SCM made with ATS, where the porosity is decreased by replacing 100% NA with 100% ATS. However, the influence of Vp/Vs was very strong. This produced a large decrease in the porosity when Vp/Vs = 0.8. The use of the dosages of groups 2 and 3 caused the APW to remain similar to the reference mixtures [51].

Figure 12 shows the results obtained for water absorption (%) for all the studied SCMs. Water absorption decreased compared with the reference mixtures as the percentage of substitution increased, except for 50ATS-1, where it increased slightly. These results are in accordance with those obtained using the APW (Figure 11) and dry bulk density (Figure 10). As the density increased, porosity and water absorption decreased.

#### 3.3.4. Shrinkage and Mass Loss

**Figure 13:**
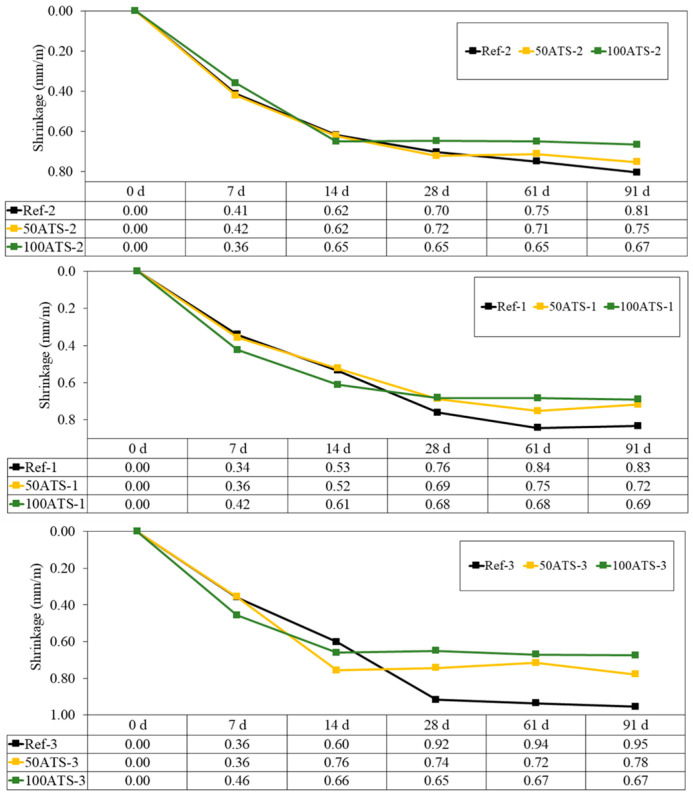
Shrinkages of all groups of studied mixtures (Ref, 50ATS, and 100ATS).

Figure 13 shows the evolution up to 91 d of the dimensional stability (shrinkage in mm/m) of all the studied groups of hardened mortars. Shrinkage decreased when NA was replaced with 50ATS and 100ATS aggregates in all the groups of mortars. High shrinkage was observed during the first 14 d; however, after 28 d, the measurements stabilised.

Mixtures with ATS absorb more water than those with NA; hence, these mixtures have less water available for typical cement hydration reactions in mortar. Water availability defines shrinkage; therefore, the increase in shrinkage in the Ref mixtures was primarily related to the higher available water content. These results agree with the dry consistency obtained for 50ATS and 100ATS in Table 4, where the values of the parameters Gm and Rm decreased at increasing ATS percentages. Lertwattanaruk et al. [40] studied shrinkage in different mortars in which OPC was replaced (replacements up to 20%) with four types of ground seashells (mussel, clam, cockle, and oyster shells), and demonstrated that all mortars manufactured with ground seashells exhibited a lower shrinkage than those of the reference mortars. This was attributed to the large pore segmentation of seashell particles, which increased the number of nucleation sites inserted in the voids between the cement particles, densified the internal structure, and decreased shrinkage.

**Figure 14:**
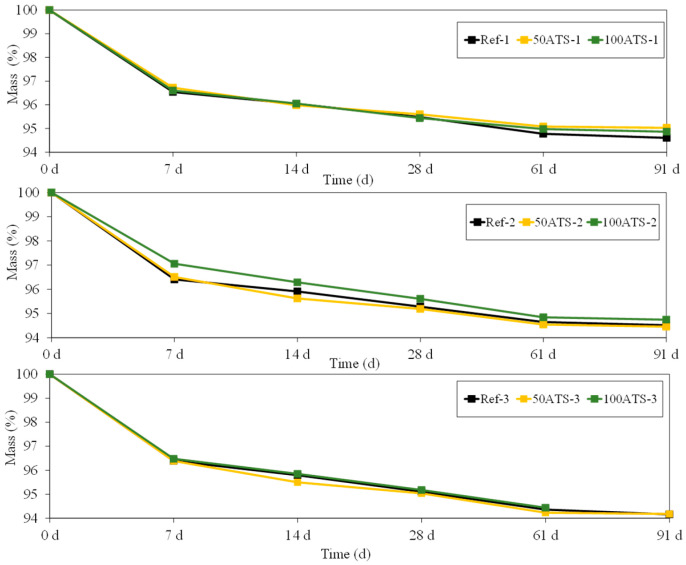
Mass (%) of all groups of studied mixtures (Ref, 50ATS, and 100ATS).

Figure 14 shows the evolution of mass (%) of all the groups of studied mortar up to 91 d. The mass loss is usually associated with water loss. This water loss coincides with the results obtained for shrinkage (Figure 13); as the amount of water in the mixture decreases (Table 2), the shrinkage decreases, thus showing that it is a drying shrinkage.

#### 3.3.5. MIP

**Figure 15:**
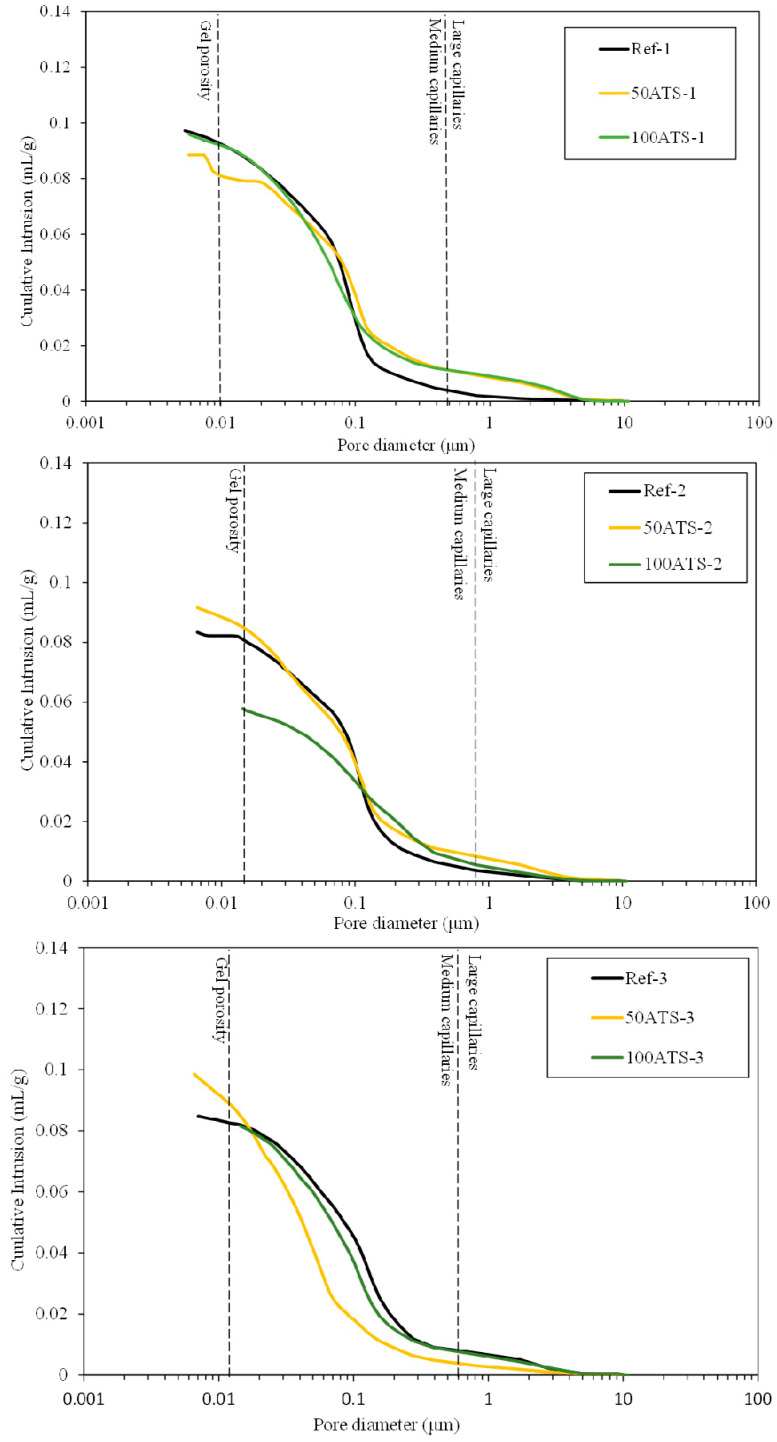
Cumulative pore volumes of the Reference, 50ATS, and 100 ATS mixtures.

**Figure 16:**
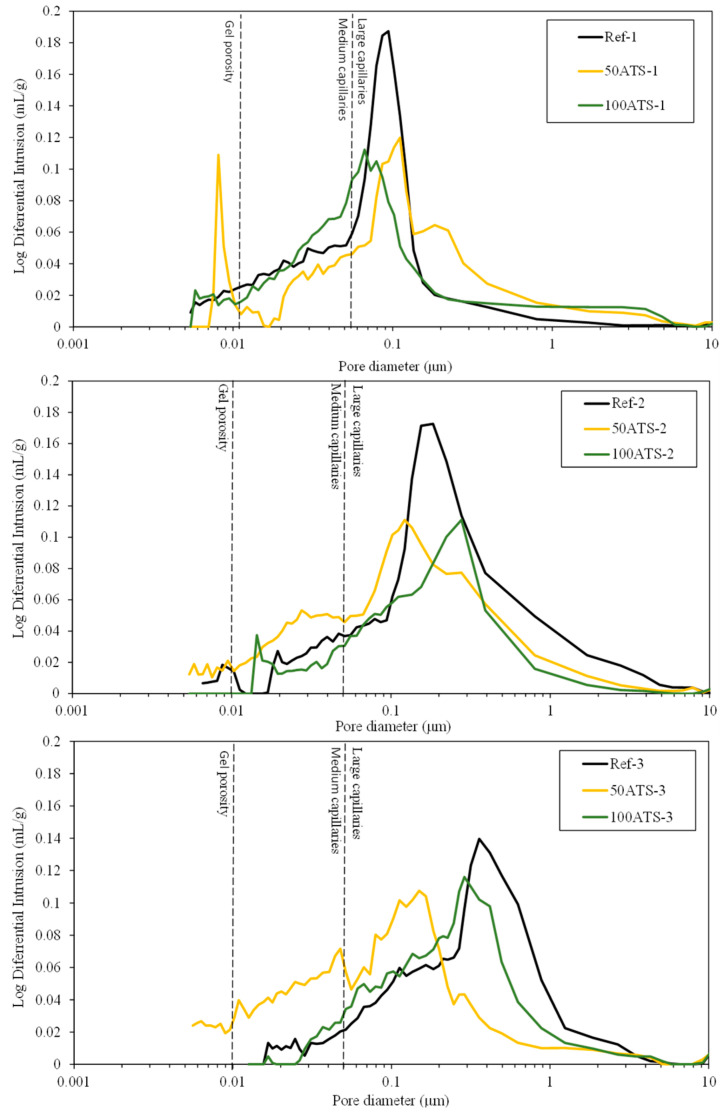
Log differential intrusion of the studied Reference and ATS aggregate SCMs.

Figure 15 shows the plot of the cumulative mercury intrusion values versus pore diameter (μm). Figure 16 shows plots of the differential mercury intrusion values versus pore diameter (μm) of the Reference and 50 and 100 ATS aggregates for all the studied mixtures. Silva et al. classified [60] the pore sizes as follows: pore diameters < 0.01 μm were designated as “gel porosity”; these were formed during the hydration process of C-S-H gels and could not be fully penetrated by mercury [18]. Medium-sized capillaries had sizes in the range of 0.01–0.05 μm. The large capillaries had sizes in the range of 0.05–1 μm. The properties affected by the “gel porosity” are shrinkage and consistency. The mechanical strength, permeability, consistency, and shrinkage properties were affected by medium capillaries. The mechanical strength and permeability properties were affected by large capillaries [60].

Ref-1, -2, and -3 recorded maximum cumulative intrusion volumes of 0.097, 0.083, and 0.085 mL/g, respectively. 50ATS-1, -2, and -3 registered maximum values of 0.089, 0.094, and 0.097 mL/g, respectively. The maximum values for 100ATS-1, -2, and -3 were 0.096, 0.058, and 0.082 mL/g, respectively. The cumulative pore volume curves did not exhibit a clear trend. It appeared that the mixtures made with the aggregate 100ATS-1, -2, and -3 had a smaller cumulative intrusion volume than the reference mixture. Liao et al. [18] studied the replacement of oyster shell aggregates with river sand in mortars, and the obtained mortars with 30% oyster shell sand exhibited the lowest porosity. The control mortars (without oyster shell sand) exhibited greater mercury intrusion than that in the oyster shell mortars, indicating a higher porosity. The cumulative pore volume curves show that the intrusion values decreased with increasing oyster shell sand content. This implies that the filling effect caused by the shells produced a poor connection between the pores, densifying the mortar structure.

Figure 16 shows the threshold diameter and provides information on the pore size at which continuous mercury intrusion begins (pore frequency). For cement-based materials, these graphs are useful for comparing similar systems and for obtaining a measure of percolation [61]. It can be observed that all the pore sizes are mainly situated in the range of large capillaries.

For Ref-1 and 50ATS-1, the maximum pore size was found to be around 0.1 μm, and for 100ATS-1 it was 0.06 μm. Ref-2 and 50ATS-2 exhibited similar pore size, approximately equal to 0.18 μm. However, for 100ATS-2, the pore size increased to 0.27 μm. Ref-3 had a pore size equal to 0.37 μm. For the mixtures of 50ATS-3 and 100ATS-3, the pore size decreased to 0.15 and 0.29 μm, respectively. Comparison of the specimens Ref-1, Ref-2, and Ref-3 showed that the pore size increased as Vp/Vs increased. This may be due to the decompensation of fine and coarse materials which produces more voids. When the substitution of natural aggregates by 50ATS occurred, the effect of the Vp/Vs ratio was very small and there was practically no variation in the pore size, except for the small number of pores in the “gel pore” range for sample 50ATS-1. When the Vp/Vs ratio was increased to 0.7, the pore size values for the sample with the 100ATS aggregate (100ATS-2) increased, in contrast to the results obtained by Liao et al. [18]. These authors observed that pore diameter and overall porosity decreased when an increased proportion of oyster shell sand was substituted (replacement up to 30%), resulting in a finer pore structure. The oyster shell sand mortar was more compact and had a finer pore system than the control mortar. Additionally, when the Vp/Vs ratio was 0.8, the pore size for the sample with 100ATS aggregate (100ATS-3) was practically unchanged. Safi et al. [59] studied the effect of incorporation of 50% and 100% waste seashell sand in SCM and concluded that, as the percentage of substitution increased, the voids were filled, owing to the elongated shape of the seashell particles; this led to the decrease of the porosity of the mortar.

The largest mercury intrusion volume in the “large capillary” region is mainly detrimental to properties such as compressive strength [62]. Accordingly, the differential mercury intrusion values for the “large capillaries” region justifies the mechanical behaviour of SCM (Figure 8).

#### 3.3.6. Water Absorption by Capillarity

**Figure 17:**
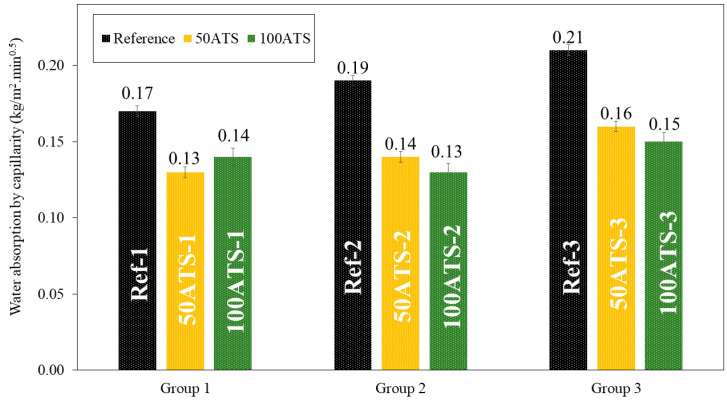
Water absorption by capillarity of all the studied groups of mixtures (Reference, 50ATS, and 100ATS).

Figure 17 shows the histograms of water absorption by capillarity of all the groups of mixtures studied after 28 d of curing. Water absorption by capillarity decreased when the natural aggregates were replaced by the ATS aggregates. The percentages of decrease compared with the reference mixtures were of 23.53%, 26.32%, and 23.81% (for 50ATS-1, 50ATS-2, and 50ATS-3, respectively), and of 17.65%, 31.58%, and 28.57% (for 100ATS-1, 100ATS-2, and 100ATS-3, respectively).

Water absorption by capillarity is related to the durability of mortars because it controls the moisture and soluble salts that reach the interior mortar parts. Therefore, lower water absorption is a positive property. The SCM manufactured with seashells absorbs a much lower water content than that absorbed by the reference mixtures. Martínez-García et al. [63] studied the water absorption capillarity in mortars manufactured with mussel shell sand and found that water absorption decreased as the percentage of seashell replacement increased. These authors related water absorption by capillarity with pore diameter; when the pore diameter decreases (pore sizes ranging between 0.1 and 1 μm), the force of water absorption increases. These results are consistent with those obtained using MIP. The reference mixtures had smaller pore sizes, which led to higher capillary absorption.

## 4. Analysis of Results

The feasibility of the use of milled ATS seashells as recycled fine aggregate in a self-compacting mortar was experimentally evaluated. The following results were obtained:

- The chemical composition of the ATS seashells was mainly calcareous (96.81%), and the mineralogical phases identified were aragonite and vaterite; both are polymorphs of CaCO_3_.
- Three groups of self-compacting mortars were manufactured by modifying the powder/sand ratios (Vp/Vs) to 0.6, 0.7, and 0.8. For each of these groups, three replacement ratios by volume (0%, 50%, and 100%) of natural limestone sand with recycled fine aggregate from ATS were studied.
- The study of the workability of self-compacting mortars by means of the V-funnel and spread tests concluded that the fluidity decreased as the incorporation of ATS aggregate increased, mainly owing to the higher water absorption of the shells and the angular and irregular shape of ATS.
- XRD showed that the incorporation of the ATS aggregate did not influence the formation of new phases with respect to the reference mortars made with limestone aggregate. Based on TGA/DTA, it was found that the amount of portlandite phase Ca(OH)_2_ increased with the incorporation of ATS. This was owing to the increased porosity of the shell particles, which increased the number of nucleation sites available for cement reactions.
- The mechanical strength was slightly reduced as the ATS increased in the self-compacting mortars due to the particle ATS morphology and higher water absorption compared with those of natural sand. Although the compressive and flexural strengths decreased, they remained similar to the reference values. It seems that the mixture belonging to group 2 (powder/sand ratio of 0.7) performed the best because the amounts of coarse and fine particles were balanced.
- The microstructure of self-compacting mortar obtained by MIP revealed that all the pore sizes were in the range of large capillaries. All the tested samples showed that the porosity increased as a function of the Vp/Vs ratio; additionally, the incorporation of ATS increased, which may be related to the shape of ATS. ATS improved the pore size by reducing large capillaries and obstructing connections between pores, resulting in a denser arrangement. The number of large capillaries, which are prone to cracking, was reduced.
- The self-compacting mortars with ATS aggregates led to a lower drying shrinkage than those of the mortars made with natural aggregates. This could be due to the higher water absorption of the shell particles in which the free-water content available in the mortar was lower.
- The water absorption by the capillaries was lower in mortars containing ATS aggregates, which improved their durability properties.

## 5. Conclusions

This study aims to investigate the effect of substituting calcareous natural aggregate (NA) with recycled aggregate from ATS to test its viability in SCM production. The use of ATS seashells as aggregate in self-compacting mortars achieves the following advantages:

- Environment sustainability: the incorporation of seashell waste helps reduce environmental pollution and encourages recycling, reducing the need of natural aggregate.
- Improved durability properties: shrinkage, water absorption by capillarity.
- Cost-effective: the use of ATS seashell waste will reduce material costs by replacing natural aggregates.

It can be concluded that the incorporation of milled ATS as a fine aggregate in self-compacting mortars is a viable alternative that promotes the circular economy in the construction sector, providing a second opportunity for the use of the waste from the bivalve mollusc canning industry and avoiding its disposal in landfills and the extraction of natural quarry sand.

The following suggestions are recommended for future work:

- Analysis of the thermal, acoustic, and electrical insulation of mortars with ATS seashells, recognising the potential improvements derived from the inclusion of these wastes.
- Use as a calcium additive in fly ash for alkaline activation due to the amount of calcium contained in seashells.
- Durability analyses could be carried out, such as resistance to carbonation, chloride and sulphate attack, freeze–thaw cycles, and fire exposure simulations.
- Analysis of the economic and environmental assessment of mortars made from ATS seashell waste, using life cycle analysis (LCA).

## Figures and Tables

**Table 1:** Basic physical parameters of the aggregates used in this study.

Aggregate	Skeletal Density ρ (g/cm^3^)	Water Absorption (%)
Natural aggregate 0/3 (NA-0/3)	2.62	1.78
Natural aggregate 0/6 (NA-0/6)	2.64	2.40
*Acanthocardia tuberculate* seashell (ATS) aggregate	2.72	2.19

**Table 2:** Nomenclature, mixing proportions (kg/m^3^), and self-compactability parameters of mortars.

		Group 1			Group 2			Group 3		
		Ref-1	50ATS	100ATS	Ref-2	50ATS	100ATS	Ref-3	50ATS	100ATS
Powders	CEM	490.9	490.9	490.9	526.3	526.3	526.3	557	557	557
	Limestone filler (LF)	300.5	300.5	300.5	322.2	322.2	322.2	341	341	341
Fine aggregates	NA-0/3	618.1	309	0	568.1	284	0	526	263	0
	NA-0/6	622.8	311.4	0	572.4	286.2	0	530	265	0
	ATS aggregate	0	642.4	1284.7	0	590.4	1180.8	0	546.7	1093.3
	Water	240.6	240.6	240.6	258	258	258	273	273	273
Self-compactability parameters	Vp/Vs	0.6	0.6	0.6	0.7	0.7	0.7	0.8	0.8	0.8
	Vw/Vp	0.85	0.85	0.85	0.85	0.85	0.85	0.85	0.85	0.85
	Sp/p %	0.6	0.6	0.6	0.6	0.6	0.6	0.5	0.5	0.5

**Table 3:** Chemical composition of all the raw materials using X-ray fluorescence spectroscopy.

Oxides (%)	Cement (CEM)	NA-0/3	NA-0/6	ATS Aggregate	LF
Na_2_O	0.17	−	−	0.47	0.08
MgO	0.95	0.48	18.90	0.05	0.53
Al_2_O_3_	2.68	0.11	0.03	0.07	1.47
SiO_2_	11.21	0.21	0.45	0.62	1.35
P_2_O_5_	0.07	−	−	−	−
SO_3_	3.45	0.04	0.05	0.08	0.05
Cl^−^	0.13	−	0.11	0.05	−
K_2_O	0.87	0.02	0.03	0.02	−
CaO	50.37	53.51	30.08	47.99	54.89
TiO_2_	0.16	−	−	−	−
MnO_2_	0.04	−	−	−	−
Fe_2_O_3_	1.76	0.05	0.11	0.05	0.36
ZnO	0.02	−	−	−	−
SrO	0.06	0.02	−	0.16	−
CO_2_ Balance	28.07	45.77	50.23	50.43	41.27

**Table 4:** Fresh properties of the studied mixtures (Reference, 50ATS, and 100ATS).

	Group 1			Group 2			Group 3		
	Ref-1	50ATS-1	100ATS-1	Ref-2	50ATS-2	100ATS-2	Ref-3	50ATS-3	100ATS-3
Gm	4.52	4.4	4.29	9.56	7.12	5.76	7.12	5.63	4.52
Rm (s^−1^)	1.25	1.00	0.91	2.00	2.00	1.53	1.67	1.25	1.00

**Table 5:** Weight loss (%) of all groups of SCM studied in different temperature ranges.

Mixtures		Weight Loss (%)			
		25 °C–105 °C	380–470 °C	470–850 °C	850–1000 °C
Group 1	Ref-1	2.23	1.11	1.15	33.30
	50ATS-1	1.44	1.18	1.19	31.91
	100ATS-1	1.62	1.14	1.15	31.67
Group 2	Ref-2	1.34	1.17	1.24	32.70
	50ATS-2	2.33	1.40	1.22	30.39
	100ATS-2	1.39	1.20	1.45	31.22
Group 3	Ref-3	1.47	1.30	1.20	32.11
	50ATS-3	1.44	1.97	1.59	32.19
	100ATS-3	2.02	1.51	1.23	27.86

## Data Availability

Data will be made available upon request.

## References

[1] Bellei P., Torres I., Solstad R., Flores-Colen I. (2023). Potential Use of Oyster Shell Waste in the Composition of Construction Composites: A Review. Buildings.

[2] González-Caro Á., Merino-Lechuga A.M., Fernández-Ledesma E., Fernández-Rodríguez J.M., Jiménez J.R., Suescum-Morales D. (2023). The Effect of Acanthocardia Tuberculata Shell Powder as Filler on the Performance of Self-Compacting Mortar. Materials.

[3] Chen B., Peng L., Zhong H., Zhao Y., Meng T., Zhang B. (2023). Improving the Mechanical Properties of Mussel Shell Aggregate Concrete by Aggregate Modification and Mixture Design. Case Stud. Constr. Mater..

[4] Eziefula U.G., Ezeh J.C., Eziefula B.I. (2018). Properties of Seashell Aggregate Concrete: A Review. Constr. Build. Mater..

[5] Asaoka S., Yamamoto T., Kondo S., Hayakawa S. (2009). Removal of Hydrogen Sulfide Using Crushed Oyster Shell from Pore Water to Remediate Organically Enriched Coastal Marine Sediments. Bioresour. Technol..

[6] Tayeh B.A., Hasaniyah M.W., Zeyad A.M., Yusuf M.O. (2019). Properties of Concrete Containing Recycled Seashells as Cement Partial Replacement: A Review. J. Clean. Prod..

[7] Martínez-garcía C., González-fonteboa B., Martínez-abella F., López D.C. (2017). Virtual Special Issue Bio Based Building Materials. Performance of Mussel Shell as Aggregate in Plain Concrete. Constr. Build. Mater..

[8] Park W.H., Polprasert C. (2008). Roles of Oyster Shells in an Integrated Constructed Wetland System Designed for P Removal. Ecol. Eng..

[9] Richardson A.E., Fuller T. (2013). Sea Shells Used as Partial Aggregate Replacement in Concrete. Struct. Surv..

[10] Li G., Xu X., Chen E., Fan J., Xiong G. (2015). Properties of Cement-Based Bricks with Oyster-Shells Ash. J. Clean. Prod..

[11] Kuo W.T., Wang H.Y., Shu C.Y., Su D.S. (2013). Engineering Properties of Controlled Low-Strength Materials Containing Waste Oyster Shells. Constr. Build. Mater..

[12] Agbede O.I., Manasseh J. (2009). Suitability of Periwinkle Shell as Partial Replacement for River Gravel in Concrete. Leonardo Electron. J. Pract. Technol..

[13] Nakatani N., Takamori H., Takeda K., Sakugawa H. (2009). Transesterification of Soybean Oil Using Combusted Oyster Shell Waste as a Catalyst. Bioresour. Technol..

[14] Olivia M., Mifshella A.A., Darmayanti L. (2015). Mechanical Properties of Seashell Concrete. Procedia Eng..

[15] Nguyen D.H., Boutouil M., Sebaibi N., Baraud F., Leleyter L. (2017). Durability of Pervious Concrete Using Crushed Seashells. Constr. Build. Mater..

[16] Mo K.H., Alengaram U.J., Jumaat M.Z., Lee S.C., Goh W.I., Yuen C.W. (2018). Recycling of Seashell Waste in Concrete: A Review. Constr. Build. Mater..

[17] Zhang Y., Chen D., Liang Y., Qu K., Lu K., Chen S., Kong M. (2020). Study on Engineering Properties of Foam Concrete Containing Waste Seashell. Constr. Build. Mater..

[18] Liao Y., Fan J., Li R., Da B., Chen D., Zhang Y. (2022). Influence of the Usage of Waste Oyster Shell Powder on Mechanical Properties and Durability of Mortar. Adv. Powder Technol..

[19] Liao Y., Wang X., Kong D., Da B., Chen D. (2023). Experiment Research on Effect of Oyster Shell Particle Size on Mortar Transmission Properties. Constr. Build. Mater..

[20] Oh S.E., Chung S.Y., Kim K., Han S.H. (2024). Comparative Analysis of the Effects of Waste Shell Aggregates on the Material Properties of Cement Mortars. Constr. Build. Mater..

[21] Shang X., Chang J., Yang J., Ke X., Duan Z. (2022). Life Cycle Sustainable Assessment of Natural vs Artificial Lightweight Aggregates. J. Clean. Prod..

[22] EU, COM(2020)98 (2020). Communication from the Commission to the European Parliament, the Council, the European Economic and Social Committee and the Committee of the Regions. A New Circular Economy Action Plan for a Cleaner and More Competitive Europe. https://eur-lex.europa.eu/legal-content/EN/TXT/?uri=celex:52020DC0098.

[23] Billberg P. (1999). Fine Mortars Rheology in Mix Design of SCC. Proceedings of the First International RILEM Symposium on Self-Compacting Concrete, Stockholm, Sweden, 13–15 September 1999.

[24] Nepomuceno M., Oliveira L., Lopes S.M.R. (2012). Methodology for Mix Design of the Mortar Phase of Self-Compacting Concrete Using Different Mineral Additions in Binary Blends of Powders. Constr. Build. Mater..

[25] Da Silva P.R., De Brito J. (2015). Fresh-State Properties of Self-Compacting Mortar and Concrete with Combined Use of Limestone Filler and Fly Ash. Mater. Res..

[26] (2011). Cement. Part 1: Composition, Specifications and Conformity Criteria for Commo on Cements.

[27] (1999). Test for chemical properties of aggregates. Part 1: Chemical analysis.

[28] (2021). Ministerio de Transportes Movilidad y Agenda Urbana Código Estructural. Boletín Oficial Del Estado.

[29] (2012). Tests for Geometrical Properties of Aggregate. Part 1: Determination of Particle Size Distribution—Sieving Method.

[30] (2009). Tests for Mechanical and Physical Properties of Aggregates. Part 7: Determination of Particle Density and Water Absorption.

[31] EFNARC (2002). Specification and Guidelines for Self-Compacting Concrete.

[32] (2020). Methods of Test for Mortar for Mansory. Part 11: Determination of Flexural and Compressive Strength of Hardened Mortar.

[33] (2014). Concrete Durability. Test Methods. Determination of the Water Absorption, Density and Accesible Porosity for Water in Concrete.

[34] Centro de Investigación, Tecnología e Innovación de La Universidad de Sevilla (CITIUS). https://citius.us.es/web/.

[35] (2021). Mortars. Methods of Test for Hardened Mortar for Mansory. Determination of Dimensional Stability of Hardened Mortar for Mansory.

[36] (2003). Methods of Test for Mortar for Mansory. Part 18: Determination of Water Absorption Coefficient Due to Capillary Action of Hardened Mortar.

[37] Merino-Lechuga A.M., González-Caro Á., Fernández-Ledesma E., Jiménez J.R., Fernández-Rodríguez J.M., Suescum-Morales D. (2023). Accelerated Carbonation of Vibro-Compacted Porous Concrete for Eco-Friendly Precast Elements. Materials.

[38] Mohit M., Haftbaradaran H., Riahi H.T. (2023). Investigating the Ternary Cement Containing Portland Cement, Ceramic Waste Powder, and Limestone. Constr. Build. Mater..

[39] Djobo Y.J.N., Elimbi A., Dika Manga J., Djon Li Ndjock I.B. (2016). Partial Replacement of Volcanic Ash by Bauxite and Calcined Oyster Shell in the Synthesis of Volcanic Ash-Based Geopolymers. Constr. Build. Mater..

[40] Lertwattanaruk P., Makul N., Siripattarapravat C. (2012). Utilization of Ground Waste Seashells in Cement Mortars for Masonry and Plastering. J. Environ. Manag..

[41] Olivia M., Oktaviani R. (2017). Ismeddiyanto. Properties of Concrete Containing Ground Waste Cockle and Clam Seashells. Procedia Eng..

[42] (2003). JCPDS, Joint Committee on Power Diffraction Standard-International Centre for Diffraction. https://pubs.acs.org/doi/10.1021/ac60293a779.

[43] Wang J., Liu E., Li L. (2019). Characterization on the Recycling of Waste Seashells with Portland Cement towards Sustainable Cementitious Materials. J. Clean. Prod..

[44] Suarez-Riera D., Merlo A., Lavagna L., Nisticò R., Pavese M. (2021). Mechanical Properties of Mortar Containing Recycled Acanthocardia Tuberculata Seashells as Aggregate Partial Replacement. Bol. Soc. Esp. Ceram. y Vidr..

[45] Suescum-Morales D., Fernández-Ledesma E., González-Caro Á., Merino-Lechuga A.M., Fernández-Rodríguez J.M., Jiménez J.R. (2023). Carbon Emission Evaluation of CO_2_ Curing in Vibro-Compacted Precast Concrete Made with Recycled Aggregates. Materials.

[46] Suescum-Morales D., Silva R.V., Bravo M., Jiménez J.R., Fernández-Rodríguez J.M., de Brito J. (2022). Effect of Incorporating Municipal Solid Waste Incinerated Bottom Ash in Alkali-Activated Fly Ash Concrete Subjected to Accelerated CO_2_ Curing. J. Clean. Prod..

[47] Kalinowska-Wichrowska K., Pawluczuk E., Bołtryk M., Jimenez J.R., Fernandez-Rodriguez J.M., Morales D.S. (2022). The Performance of Concrete Made with Secondary Products—Recycled Coarse Aggregates, Recycled Cement Mortar, and Fly Ash–Slag Mix. Materials.

[48] Barros M.C., Bello P.M., Bao M., Torrado J.J. (2009). From Waste to Commodity: Transforming Shells into High Purity Calcium Carbonate. J. Clean. Prod..

[49] Mohamed M., Rashidi N.A., Yusup S., Teong L.K., Rashid U., Ali R.M. (2012). Effects of Experimental Variables on Conversion of Cockle Shell to Calcium Oxide Using Thermal Gravimetric Analysis. J. Clean. Prod..

[50] Perić J., Vueak M., Krstulovib R., Breeevib L., Kralj D. (1996). Phase Transformation of Calcium Carbonate Polymorphs. Thermochim. Acta.

[51] Yang E.I., Yi S.T., Leem Y.M. (2005). Effect of Oyster Shell Substituted for Fine Aggregate on Concrete Characteristics: Part I. Fundamental Properties. Cem. Concr. Res..

[52] Lozano-Lunar A., Dubchenko I., Bashynskyi S., Rodero A., Fernández J.M., Jiménez J.R. (2020). Performance of Self-Compacting Mortars with Granite Sludge as Aggregate. Constr. Build. Mater..

[53] Hamada H.M., Abed F., Tayeh B., Al Jawahery M.S., Majdi A., Yousif S.T. (2023). Effect of Recycled Seashells on Concrete Properties: A Comprehensive Review of the Recent Studies. Constr. Build. Mater..

[54] Nduka D.O., Akanbi E.T., Ojo D.O., Babayemi T.E., Jolayemi K.J. (2023). Investigation of the Mechanical and Microstructural Properties of Masonry Mortar Made with Seashell Particles. Materials.

[55] Song Q., Wang Q., Xu S., Mao J., Li X., Zhao Y. (2022). Properties of Water-Repellent Concrete Mortar Containing Superhydrophobic Oyster Shell Powder. Constr. Build. Mater..

[56] Esquinas A.R., Ramos C., Jiménez J.R., Fernández J.M., de Brito J. (2017). Mechanical Behaviour of Self-Compacting Concrete Made with Recovery Filler from Hot-Mix Asphalt Plants. Constr. Build. Mater..

[57] Suescum-Morales D., Fernández-Rodríguez J.M., Jiménez J.R. (2022). Use of Carbonated Water to Improve the Mechanical Properties and Reduce the Carbon Footprint of Cement-Based Materials with Recycled Aggregates. J. CO_2_ Util..

[58] Suescum-Morales D., Cantador-Fernández D., Ramón Jiménez J., María Fernández J. (2021). Potential CO_2_ Capture in One-Coat Limestone Mortar Modified with Mg_3_Al–CO_3_ Calcined Hydrotalcites Using Ultrafast Testing Technique. Chem. Eng. J..

[59] Safi B., Saidi M., Daoui A., Bellal A., Mechekak A., Toumi K. (2015). The Use of Seashells as a Fine Aggregate (by Sand Substitution) in Self-Compacting Mortar (SCM). Constr. Build. Mater..

[60] Silva D.A., John V.M., Ribeiro J.L.D., Roman H.R. (2001). Pore size distribution of hydrated cement pastes modified with polymers. Cem. Concr. Res..

[61] Abell A.B., Willis K.L., Lange D.A. (1999). Mercury intrusion porosimetry and image analysis of cement-based materials. J. Colloid. Interface Sci..

[62] Ortega-López V., Fuente-Alonso J.A., Santamaría A., San-José J.T., Aragón Á. (2018). Durability Studies on Fiber-Reinforced EAF Slag Concrete for Pavements. Constr. Build. Mater..

[63] Martínez-García C., González-Fonteboa B., Carro-López D., Martínez-Abella F. (2020). Effects of Mussel Shell Aggregates on Hygric Behaviour of Air Lime Mortar at Different Ages. Constr. Build. Mater..

